# In vitro activity of ivermectin against *Staphylococcus aureus* clinical isolates

**DOI:** 10.1186/s13756-018-0314-4

**Published:** 2018-02-20

**Authors:** Shoaib Ashraf, Umer Chaudhry, Ali Raza, Debasri Ghosh, Xin Zhao

**Affiliations:** 1 Department of Animal Science, McGill University, Sainte-Anne-de-Bellevue, Canada; 2 Department of Parasitology, University of Veterinary and Animal Sciences, Lahore, Pakistan; 3 Department of Clinical Medicine and Surgery, University of Agriculture Faisalabad, Faisalabad, Pakistan

**Keywords:** Anthelmintics, Ivermectin, *Staphylococcus aureus*, Time-kill kinetics

## Abstract

**Background:**

Ivermectin is an endectocide against many parasites. Though being a macrocyclic lactone, its activity against bacteria has been less known, possibly due to the fact that micromolar concentrations at tissue levels are required to achieve a therapeutic effect. Among pathogenic bacteria of major medical significance, *Staphylococcus aureus* cause a number of diseases in a wide variety of hosts including humans and animals. It has been attributed as one of the most pathogenic organisms. The emergence of methicillin resistance has made the treatment of *S. aureus* even more difficult as it is now resistant to most of the available antibiotics. Thus, search for alternate anti-staphylococcal agents requires immediate attention.

**Methods:**

Twenty-one clinical isolates of *S. aureus* were isolated from bovine milk collected from Lahore and Faisalabad Pakistan. Different anthelmintics including levamisole, albendazole and ivermectin were tested against *S. aureus* to determine their minimum inhibitory concentrations. This was followed-up by growth curve analysis, spot assay and time-kill kinetics.

**Results:**

The results showed that ivermectin but not levamisole or albendazole exhibited a potent anti-staphylococcal activity at the concentrations of 6.25 and 12.5 μg/ml against two isolates. Interestingly, one of the isolate was sensitive while the other was resistant to methicillin/cefoxitin.

**Conclusions:**

Our novel findings indicate that ivermectin has an anti-bacterial effect against certain *S*. *aureus* isolates. However, to comprehend why ivermectin did not inhibit the growth of all Staphylococci needs further investigation. Nevertheless, we have extended the broad range of known pharmacological effects of ivermectin. As pharmacology and toxicology of ivermectin are well known, its further development as an anti-staphylococcal agent is potentially appealing.

## Background

Anthelmintics are drugs used for controlling helminthes. The major classes of broad spectrum anthelmintics include benzimidazoles (BZ) (e.g. albendazole (ALB)), imidathiozoles (e.g. levamisole (LEV)), tetrahydropyrimidines (e.g. pyrantel), and macrocyclic lactones (MLs) (e.g. ivermectin (IVM)). These four classes of anthelmintics have different modes of action. BZs act by binding to the growing ends of microtubules and thus prevent addition of new β-tubulin dimers. As a result, the microtubules shorten and ultimately disappear thus disrupting essential functions which leads to death of the parasite [1]. Imidathiozoles and tetrahydropyrimidines target the nicotinic receptors i.e. they bind to acetylcholine-gated cation channels as agonists at the neuromuscular receptor and cause spastic paralysis of the parasite leading to its death [2, 3]. MLs act on the ligand-gated-chloride channels (glutamate-gated chloride channels) and cause the channels to irreversibly open by binding to the site other than the glutamate binding site and this leads to a permanent hyperpolarization and paralysis of the cells and results in death of the parasite [4].

Due to limited numbers of drugs entering the market and emergence of drug resistance to major classes of anti-bacteria, scientists have been investigating known drugs with previous unknown anti-bacterial activities. Some groups have recently reported anti-bacterial activity of various anthelmintics against different bacteria. For example, Imperi et al. [5] have proposed that niclosamide which is an anthelmintic used against tapeworms could reduce the pathogenicity of *Pseudomonas aeruginosa*. Similarly, Rajamuthiah et al. [6] have reported an anti-staphylococcal activity of closantel which belongs to the salicylanilide group of anthelmintics. Furthermore, the activity of niclosamide and oxycolzanide (a flukicidal) against *S. aureus* has also been reported [7]. Finally, Gooyit and Janda [8] have reported that the salicylanilide anthelmintics (closantel, rafoxinide, niclosamide, oxyclozanide) were also effective against *Clostridium difficile*.

IVM has shown activities against a broad range of host species and thus it has earned the title of a wonder drug [9, 10]. This enigmatic multifaceted drug continues to surprise and exceed expectations. For example, IVM at sub-lethal concentrations has been reported to have an anti-plasmodial activity by inhibiting sporogony of *Plasmodium falciparum* [11]. IVM also acts as an anti-viral agent against flavivirus by inhibiting its replication through targeting the N3 helicase activity [12]. In addition, an anti-mycobacterial activity of IVM has also been identified against *Mycobacterium tuberculosis* and *Mycobacterium ulcerans* [13, 14]. Sharmeen et al. [15] found that IVM killed leukemic cells by a chloride dependent membrane hyperpolarization. Recently, anti-mitotic activities of IVM have also been reported i.e. by binding to the tubulins and consequently altering the polymerization equilibrium and leading the cells into mitotic arrest [16, 17]. However, despite being a macrocyclic lactone, its activity against bacteria has been less known, possibly because micromolar concentrations are required at tissue levels to achieve a therapeutic effect.

*Staphylococci* are gram-positive spherical bacteria that contain many species. It has been suggested that 30% of the world population are silent carriers of *S. aureus* without any symptoms [18]. However, *S. aureus* can cause a wide range of diseases from skin and soft-tissue infection to life-threatening diseases in humans and is also the leading cause of bacteremia [19]. In the dairy industry, *S. aureus* is also one of the most common bacteria causing mastitis [20]. β-lactams have been widely used for the treatment of *S. aureus* infections. Due to emergence of resistance to β-lactams, methicillin, a semi-synthetic penicillinase resistant β-lactam, was produced and introduced into the market in 1959. Unfortunately, it became resistant just two-years after its introduction [21]. Currently, many *S. aureus* clinical isolates are resistant to almost all the available antibiotics and the term methicillin resistant *Staphylococcus aureus* (MRSA) and vancomycin resistant *S. aureus* are heard all around the globe [22]. The challenges related to the MRSA type alone can be visualized as the mortality associated with invasiveness of MRSA has gone up to 20% [23]. Hence, in 2017, the World Health Organization has put *S. aureus* in the list of microorganisms which immediately need new antibiotics for its treatment. The main problem with MRSA phenotype is that the organisms that are methicillin resistant are often resistant to most of the known antibiotics.

In an effort to search for alternative treatment of *S. aureus* infections, in the present study, we screened three known anthelmintics (IVM, ALB, and LEV) and found that IVM but not ALB or LEV had a potent anti-bacterial activity against *S. aureus* species.

## Methods

### Bacterial isolates

Twenty-one *S. aureus* isolates were used in this study. The isolates were isolated from clinical mastitis cases from Lahore and Faisalabad, Pakistan. Among them 11 isolates were methicillin sensitive whereas 10 were methicillin resistant. The *S. aureus* isolates were grown in the Mueller Hinton (MH) broth or tryptic soya broth (TSB) (Sigma Aldrich, Canada) at 37 °C.

### Determination of minimum inhibitory concentrations (MICs)

To determine the minimum inhibitory concentrations (MICs) for the *S. aureus* isolates, 5 X 10^5^ CFU/ml cells were inoculated into the Mueller Hinton (MH) broth (Sigma Aldrich, Canada). One hundred fifty μl of the bacterial solution was dispensed into each well of 96-well round bottom microtiter plates (Sarstedt, Canada). The test compounds IVM, ALB, LEV, cefoxitin (FOX) and dimethyl sulfoxide (DMSO) (as a solvent control) (Sigma Aldrich) were serially diluted 2-fold in the MH broth. IVM and LEV dilutions were from 100 to 1.56 μg/ml, while ALB was from 50 to 0.78 μg/ml and FOX was from 128 to 2 μg/ml. The MH broth with the bacterial suspension and test compounds were incubated for 18 h at 35 °C. The MICs were determined by examining visible bacterial growth with naked eyes.

### Effects of IVM on growth curves

To determine the effects of IVM on growth curves, glycerol stocks of MSSA (O9) and MRSA (P22) isolates were grown overnight in the tryptic soya broth (TSB) (Sigma Aldrich) at 35 °C. A loop full of overnight cultures were streaked on the MH agar and the plates were further incubated for 24 h at 35 °C. After 24 h, a 0.5 McFarland culture was prepared in the MH broth. The bacteria were diluted to a final concentration of 5 X 10^5^ CFU/ml. Different test compounds including IVM, ALB, LEV, and FOX were then added at a concentration of 50, 50, 50 or 16 μg/ml, respectively. In addition,, FOX was also added at a concentration of 32 μg/ml. DMSO was used as a solvent control. The bacteria were then grown up to 14 h at 35 °C and 1 ml sample was taken every 2 h. The optical density (O.D) was measured at 600 nm. Among the 20 isolates tested, the two *S. aureus* isolates against which IVM had a more potent activity were used in further studies. For two specific *S. aureus* isolates (P22 (MRSA) and O9 (MSSA)), IVM was then serially titrated at concentrations of 100, 50, 25, 12.5, 6.25, 3.125 and 1.56 μg/ml and O.D values were taken at 2 h intervals for a total of 14 h. All the bacteria were grown at 200 rpm at 35 °C.

### Spot assay

The 0.5 McFarland culture of both isolates was prepared and further diluted (as described above) to get final concentrations of 5 X 10^5^ CFU/ml. IVM was added at concentrations of 0, 1.56, 3.125, 6.25, 12.5, 25, 50, and 100 μg/ml. After 12 h incubation at 35 °C, 5 μl bacteria from both isolates were spotted on the MH agar. The plates were then incubated at 35 °C for 24 h. Pictures were then taken using a transilluminator FBTIV-816, Kodak EDAS 290 (Fischer Scientific, Canada).

### Time kill-kinetics

For further confirmation of the above results, bacterial suspension of 0.5 McFarland was prepared for both isolates from freshly prepared overnight cultures as described above. 0.5 McFarland culture for both *S. aureus* (MSSA and MRSA) isolates was transferred to the MH broth to attain a final concentration of 5 X 10^5^ CFU/ml. Then IVM was added at concentrations of 1/4X(MIC), 1/2X(MIC), 0X(MIC), 1X(MIC), 2X(MIC), 4X(MIC), 8X(MIC) and 12X(MIC) for both isolates. The tubes were then incubated at 35 °C and 100 μl samples were taken at 0, 2, 4, 8 and 12 h. The aliquots were then plated onto MH agar plates to assess viable bacteria by CFU counting. After 24 h of incubation at 35 °C, plates were examined for growth.

## Results

### Minimum inhibitory concentrations of different drugs against *S. aureus*

As shown in Table 1, MICs for IVM were 6.25 μg/ml and 12.5 μg/ml for O9 (MSSA) and P22 (MRSA), respectively. The bacterial growth was not inhibited by ALB up to 50 μg/ml or by LEV up to 100 μg/ml. The limited solubility of ALB and LEV did not allow us to go beyond 50 and 100 μg/ml, respectively. The MIC of FOX was 4 μg/ml for O9 and 16 μg/ml for P22.

**Table 1:** Minimum inhibitory concentration (MIC)

	MIC μg/ml	
	P22 (MRSA)	O9 (MSSA)
Ivermectin (IVM)	12.5	6.25
Levamisole (LEV)	ND	ND
Albendazole (ALB)	ND	ND
Cefoxitin (FOX)	32	4

*ND* Not detectable up to 100 μg/ml for LEV and 50 μg/ml for ALB

### Inhibition of *S. aureus* growth by IVM as evidenced by growth curves

**Fig. 1:**
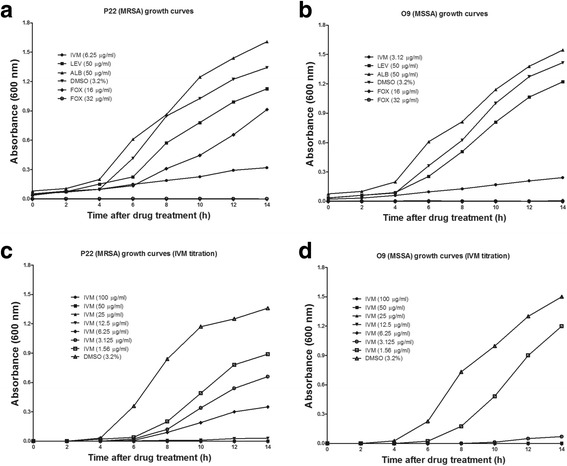
Growth curves of the P22 (MRSA) (**a**, **c**) and O9 (MSSA) (**b**, **d**) isolates in the presence of different drugs. Ivermectin (IVM), levamisole (LEV), albendazole (ALB), dimethyl-sulphoxide (DMSO) and cefoxitin (FOX) were used as test compounds at fixed concentrations

The growth curve analysis demonstrated that ALB, LEV, and DMSO did not have any effect on the growth of both *S. aureus* isolates (Fig. 1a, b), whereas IVM reduced the growth of both isolates. For FOX, O9 showed no growth at both concentrations tested whereas P22 showed growth at 16 μg/ml but no growth was observed at 32 μg/ml. The 2-fold IVM titration from 100 to 1.56 μg/ml demonstrated that IVM inhibited bacterial growth at concentrations above 3.125 μg/ml for O9 and 6.25 μg/ml for the P22 isolates (Fig. 1c, d).

**Fig. 2:**
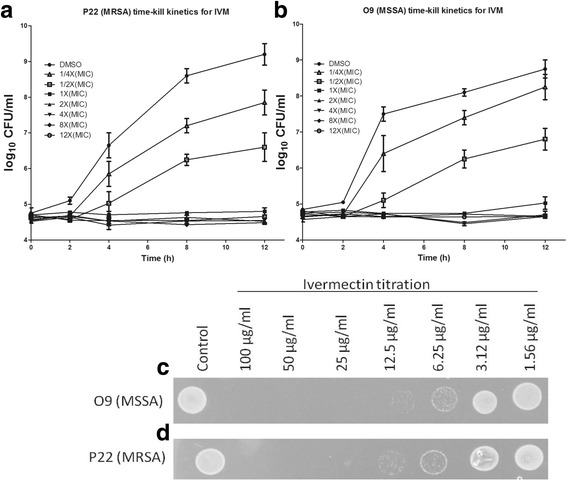
Time-kill kinetics and the spot assay results for P22 (MRSA) (**a**, **d**) and O9 (MSSA) (**b**, **c**) respectively. The error bars were standard error of the means for three independent experiments for the time-kill kinetics. The spots are representative of three independent experiments

### Inhibition of *S. aureus* growth by IVM as shown in the spot assay and time-kill kinetics

The spot assay showed no growth in both isolates beyond their MICs representing absence of any bacterial spots at concentration of 12.5 μg/ml for the O9 (MSSA) and 25 μg/ml for the P22 (MRSA) isolates (Fig. 2c, d). The results of time-kill kinetics were similar to those of the spot assay and IVM showed reduction in CFU/ml as compared to the control (DMSO) which suggested that bacterial growth was completely inhibited by IVM at concentrations of 1X(MIC) and above for both isolates (Fig. 2a, b).

## Discussion

IVM is a drug used against helminthes of human and veterinary importance. To our knowledge, this study is a maiden attempt reporting the activity of IVM against *S. aureus* clinical isolates. Interestingly, one of the isolates had a methicillin sensitive and the other had a methicillin resistant phenotype. The MICs for IVM against the MSSA (O9) and MRSA (P22) isolates were 6.25 and 12.5 μg/ml, respectively. The findings of growth curve experiments were in correlation with the MICs as there was no increase in the O.D values beyond 3.125 μg/ml and 6.25 μg/ml for both isolates. The spot assay also showed similar results to the MIC, and growth curve experiments as there was complete inhibition of growth on the bacterial spots beyond the MICs. Time-kill kinetics was also in complete agreement with the other data and showed complete inhibition of bacterial growth beyond 1X(MIC) which means the CFU did not go above 5 X 10^5^ CFU/ml. However, the isolates were not killed even when the concentration of IVM was increased up to 12X (MIC). In contrast, the number of bacteria increased when IVM concentrations were tested below the MICs.

The findings from the time-kill kinetics data indicated that the effects of IVM was probably bacteriostatic rather than bactericidal. When IVM acts as a bacteriostatic agent, it would be beneficial as the bacteriostatic antibiotics could circumvent some problems related to bactericidal drugs. For example, when the bactericidal drugs kill the bacteria, endotoxins are released which may be toxic to the host, whereas, in the case of bacteriostatic drugs, the bacterial growth is inhibited enabling the host to elicit protective immunity and thus results in immunological clearance of the bacteria [24]. Torres et al. [25] reported that IVM was effective against biofilm formation by *S. aureus*. Our results extend these previous findings about antibacterial efficacy of IVM against *S. aureus*.

*S. aureus* infections have been reported all over the world. The treatment of infections associated to *S. aureus* infections has become difficult because of the emergence of antibiotic resistance. Hence, new drugs other than conventional antibiotics should be immediately introduced to tackle the problem and minimize the selection pressure on resistant isolates. To that end, we tested antibacterial activities of anthelmintics on *S. aureus* and found that IVM showed antibacterial activities against 2 isolates of 20 *S aureus* isolates. It would be of interest to further investigate the reason why other isolates were not sensitive to this drug. It is known that IVM is a good substrate of P-glycoprotein (efflux pumps) in helminthes [26, 27]. The evidence of involvement of drug efflux pumps clearly requires further investigations. IVM is used at a dose rate of 200 μg/kg body weight in humans and animals. At this dosage, the maximum plasma concentration of IVM goes up to 52 ng/ml [28]. The concentration at which the anti-staphylococcal activity of IVM was evident in this study is higher than the concentration of its current therapeutic use and perhaps this might be the reason why this mechanism has not been reported previously. However, the lethal dose 50 (LD_50_) of IVM has been reported up to 50 mg/kg [29], which suggests that IVM has a wide therapeutic index. At this dosage, IVM concentration at tissue levels is at the low micromolar range. Hence, we report anti-staphylococcal activity of IVM against MRSA and MSSA isolates at pharmacologically relevant concentrations. Given that IVM is already approved for treatment against various parasites in humans and animals, its development as a potential antimicrobial agent to kill *S. aureus*, especially MRSA, is an appealing option.

## Conclusions

The present study investigated the antibacterial effects of IVM, ALB and LEV against methicillin sensitive and methicillin resistant *S. aureus*. Among these anthelmintics, only IVM showed a potent anti-staphylococcal activity. The MICs for IVM against MSSA and MRSA isolates were 6.25 and 12.5 μg/ml, respectively. The growth curves corresponded to the MICs. The spot assay and time kill kinetics also showed similar results to the MIC, and growth curve experiments. In summary, this study is the first attempt to indicate that IVMs also has anti-bacterial efficacy against *S. aureus*. Further development of IVM as an anti-microbial agent is potentially appealing, as its pharmacokinetics and pharmacodynamics have already been studied in mammals.

## Abbreviations

ALB: Albendazole
BZ: Benzimidazole
CFU: Colony Forming Unit
DMSO: Dimethy-Sulphoxide
FOX: Cefoxitin
IVM: Ivermectin
LEV: Levamisole
MIC: Minimum Inhibitory Concentration
MRSA: Methicillin Resistant Staphylococcus aureus
MSSA: Methicillin Sensitive Staphylococcus aureus
